# Integrated transcriptomic and proteomic analysis reveals the complex molecular mechanisms underlying stone cell formation in Korla pear

**DOI:** 10.1038/s41598-021-87262-3

**Published:** 2021-04-08

**Authors:** Aisajan Mamat, Kuerban Tusong, Juan Xu, Peng Yan, Chuang Mei, Jixun Wang

**Affiliations:** Institute of Horticultural Crops, Xinjiang Academy of Agricultural Sciences, 403 Nanchang Road, Urumqi, 830091 China

**Keywords:** Cell wall, Cell fate, Plant morphogenesis, Secondary metabolism, Differentiation, Cell biology, Developmental biology, Molecular biology, Plant sciences

## Abstract

Korla pear (*Pyrus sinkiangensis* Yü) is a landrace selected from a hybrid pear species in the Xinjiang Autonomous Region in China. In recent years, pericarp roughening has been one of the major factors that adversely affects fruit quality. Compared with regular fruits, rough-skin fruits have a greater stone cell content. Stone cells compose sclerenchyma tissue that is formed by secondary thickening of parenchyma cell walls. In this work, we determined the main components of stone cells by isolating them from the pulp of rough-skin fruits at the ripening stage. Stone cell staining and apoptosis detection were then performed on fruit samples that were collected at three different developmental stages (20, 50 and 80 days after flowering (DAF)) representing the prime, late and stationary stages of stone cell differentiation, respectively. The same batches of samples were used for parallel transcriptomic and proteomic analysis to identify candidate genes and proteins that are related to SCW biogenesis in Korla pear fruits. The results showed that stone cells are mainly composed of cellulose (52%), hemicellulose (23%), lignin (20%) and a small amount of polysaccharides (3%). The periods of stone cell differentiation and cell apoptosis were synchronous and primarily occurred from 0 to 50 DAF. The stone cell components increased abundantly at 20 DAF but then decreased gradually. A total of 24,268 differentially expressed genes (DEGs) and 1011 differentially accumulated proteins (DAPs) were identified from the transcriptomic and proteomic data, respectively. We screened the DEGs and DAPs that were enriched in SCW-related pathways, including those associated with lignin biosynthesis (94 DEGs and 31 DAPs), cellulose and xylan biosynthesis (46 DEGs and 18 DAPs), S-adenosylmethionine (SAM) metabolic processes (10 DEGs and 3 DAPs), apoplastic ROS production (16 DEGs and 2 DAPs), and cell death (14 DEGs and 6 DAPs). Among the identified DEGs and DAPs, 63 significantly changed at both the transcript and protein levels during the experimental periods. In addition, the majority of these identified genes and proteins were expressed the most at the prime stage of stone cell differentiation, but their levels gradually decreased at the later stages.

## Introduction

Korla pear (*Pyrus sinkiangensis* Yü) is a native species of the Xinjiang Autonomous Region in China. It is known for its aroma, juicy flesh, and crisp texture. Increased stone cell content, which leads to the formation of rough-skin fruits, is one of the most critical factors that result in decreased fruit quality. In regular fruits, stone cells are nearly invisible, but in rough-skin fruits, the high stone cell content imparts a very gritty texture to the fruit pulp. The rough skins are invisible phenotypically on the early fruits and gradually become visible in the later stage (from late July to mid-August). Hence, it is difficult to determine the time when the pericarp roughening is actually initiated. This makes it challenging to study the growth mechanism of rough-skin formation on pear fruits. Therefore, in order to find out the stress factors that induce the formation of rough-skinned fruits, we must first understand the underlying physiological and molecular mechanisms of stone cell differentiation.

Previous studies have shown that the formation of stone cells is closely related to the synthesis, transfer, and deposition of lignin^1,2^. Apart from tracheary elements (TEs) and fibers, sclereids (stone cells) are also formed by the secondary thickening of cell walls^3^. Cellulose, hemicellulose, and lignin constitute the main components of secondary walls, and their proportions may vary among different plant species. While we understand a great deal about the genes involved in lignin synthesis and deposition during stone cell differentiation in pear fruits^4–10^, we know surprisingly little about the underlying physiological and molecular mechanisms of stone cell differentiation because cellulose and hemicellulose account for the largest percentage of secondary cell wall (SCW) biomass. Therefore, the formation of stone cells cannot be fully explained merely by the process of lignin deposition. After their biosynthesis, lignin monomers must be transported to the cell wall where they are oxidized for polymerization. Reactive oxygen species (ROS) play a vital role in this process^11–13^. In our previous work, we found that apoptosis occurs along with the differentiation of stone cells during fruit development, and the apoptosis period overlaps with the period of stone cell formation and ROS accumulation^14^. ROS is the main stress factor leading to apoptosis. Therefore, we hypothesized that the formation of stone cells might essentially be linked to the process of apoptosis triggered by ROS. H_2_O_2_ induces cell wall thickening by polymer cross-linking, leading to growth inhibition of some parenchyma cells. As a result, some parenchyma cells are forced to differentiate. However, few studies have linked the formation of stone cells to the synthesis of cell wall polymers and cross-linkages among them. To resolve this scientific problem and theoretical inference, in this study, parallel analyses of the transcriptome and proteome of Korla pear fruits at three developmental stages were carried out to identify essential regulators and pathways involved in stone cell formation. The results gained through this study will help us understand the mechanisms underlying stone cell formation in pear fruits.

## Results

### Morphological and physiological analysis

**Figure 1:**
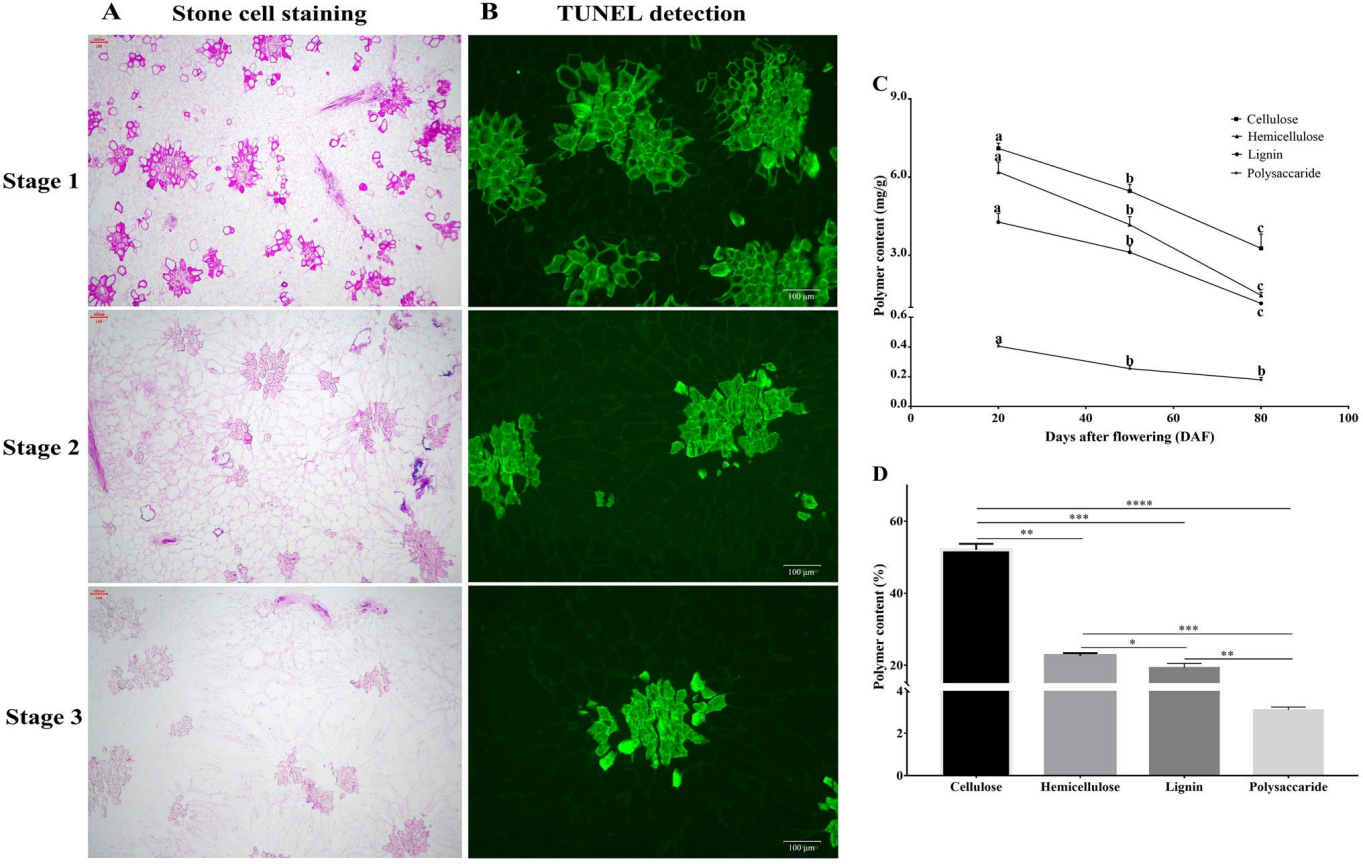
Morphological and physiological differences in Korla pear fruit pulp during the experimental time course. (**A**) Safranin staining of stone cells. Magnification: 10 × 4, Scale: 100 μm. (**B**) TUNEL detection of pear fruit cell apoptosis during the experimental time course. Magnification: 10 × 4, Scale: 100 μm. (**C**) Variation in cell wall polymers during fruit development. (D) Composition of stone cells. The bars represent the means ± SDs (n = 3).

Stone cells were isolated from rough-skin fruits at the ripening stage, and the main components were analyzed. The results revealed that the stone cells are mainly composed of cellulose (52%), hemicellulose (23%), lignin (20%), and a small amount of polysaccharides (3%) (Fig. 1D). The variation trends of these polymers, stone cell staining and cell apoptosis were then observed at three important time points of stone cell differentiation (20 DAF, 50 DAF and 80 DAF, representing the prime, late and stationary stages of stone cell differentiation, respectively). It is evident from the data that stone cell formation primarily occurred from 0 to 50 DAF, and the apoptotic period overlapped with this period (Fig. 1A, B). All the stone cell components were abundant at 20 DAF, after which the levels decreased gradually (Fig. 1C), and the trends of these components were consistent with trends of stone cell contents during fruit development. These results also indicated that the formation of stone cells could not be fully explained by lignin deposition in the secondary walls.

### Overview of transcriptomic and proteomic data

**Figure 2:**
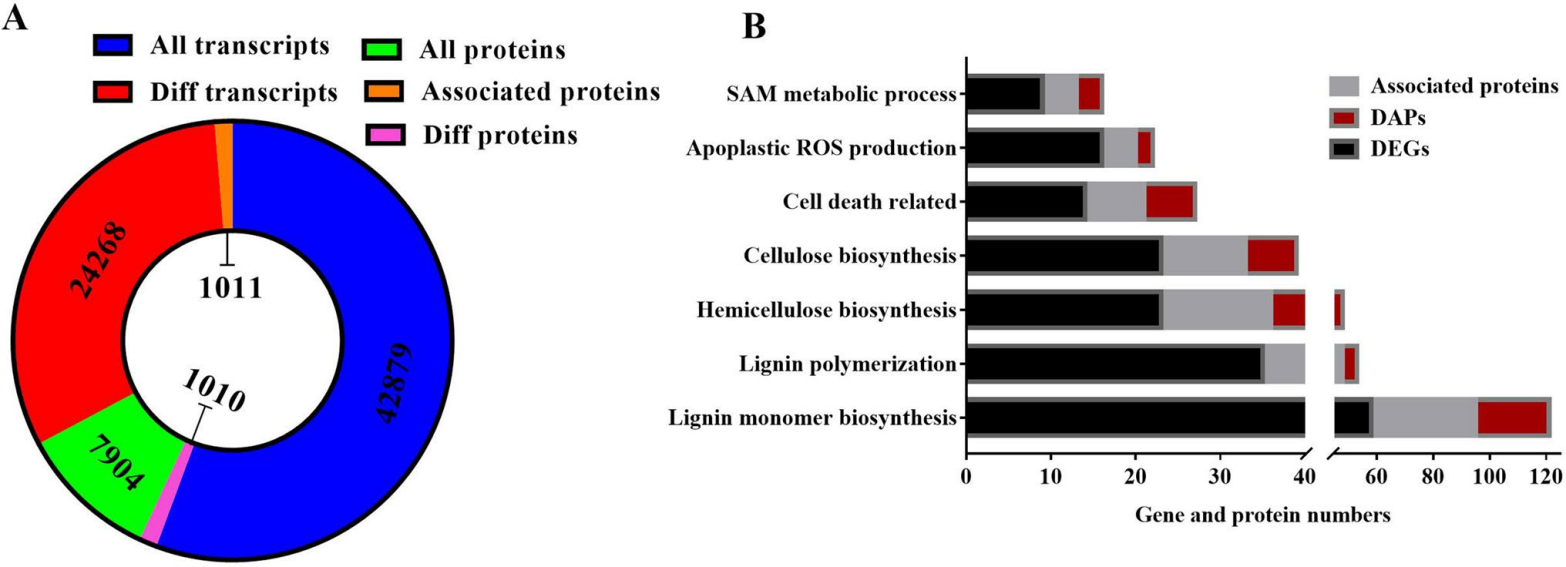
Overview of transcriptomic and proteomic data. (**A**) Circular graph showing the total genes and proteins, DEGs and DAPs, as well as associated genes and proteins identified. (**B**) Bar chart listing the pathways of interest and the number of DEGs and DAPs enriched in these pathways.

**Figure 3:**
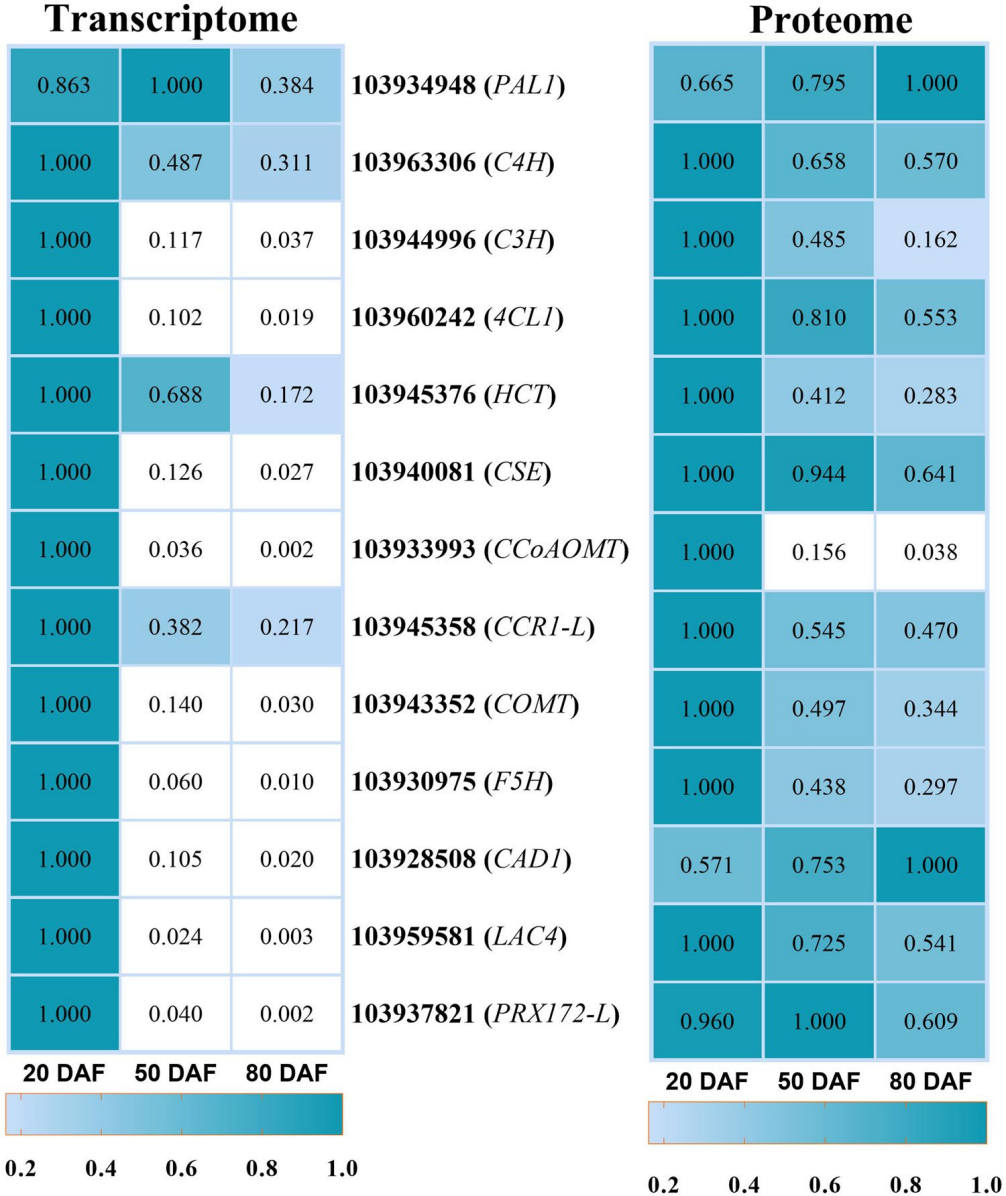
Expression of genes and proteins involved in lignin biosynthesis processes. The heat maps were produced using standardized figures that were transformed to a value between 0.0 and 1.0 by the min–max normalization method.

**Figure 4:**
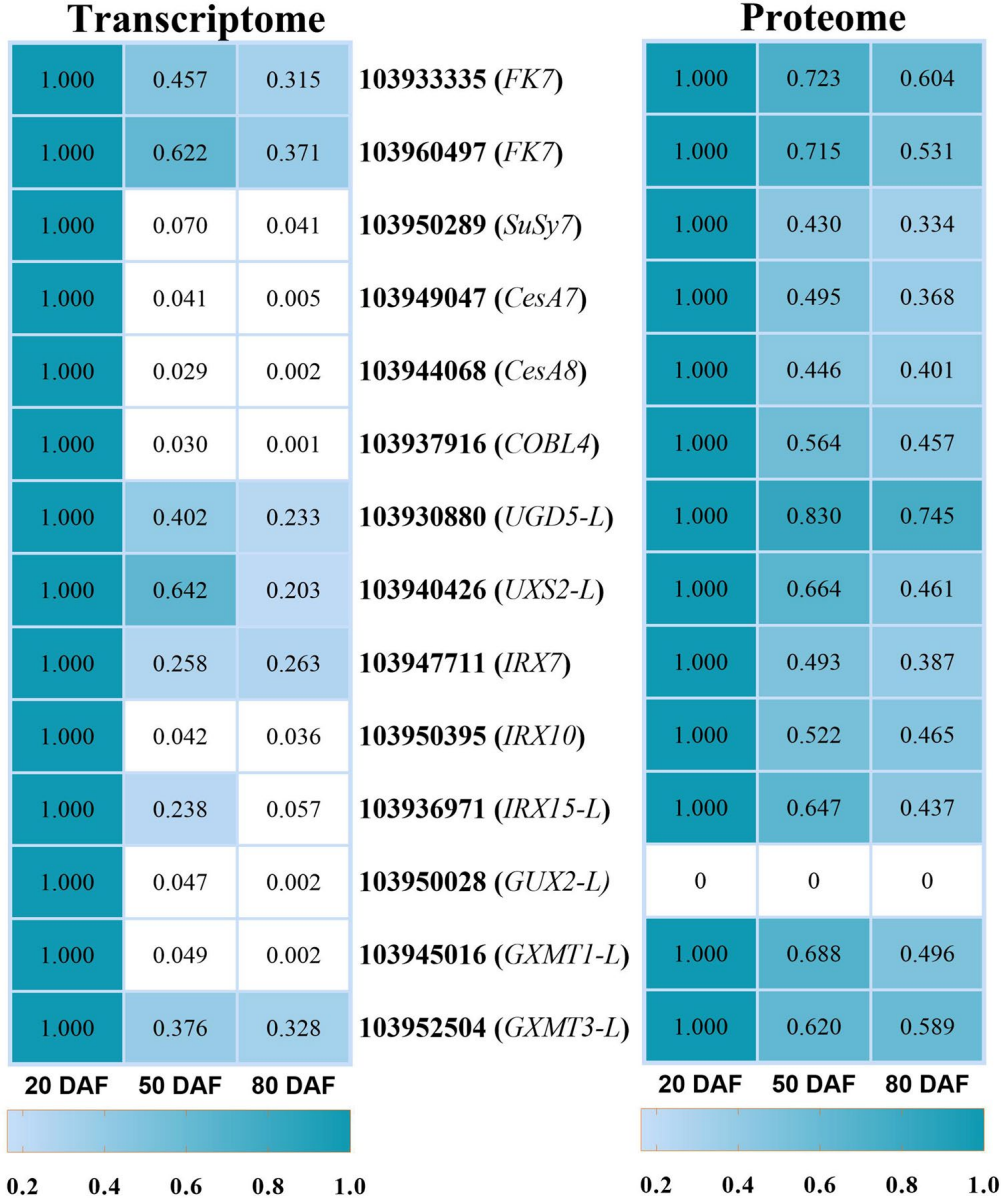
Genes and proteins involved in cellulose and xylan biosynthesis. The heat maps were produced using standardized figures that were transformed to a value between 0.0 and 1.0 by the min–max normalization method.

**Figure 5:**
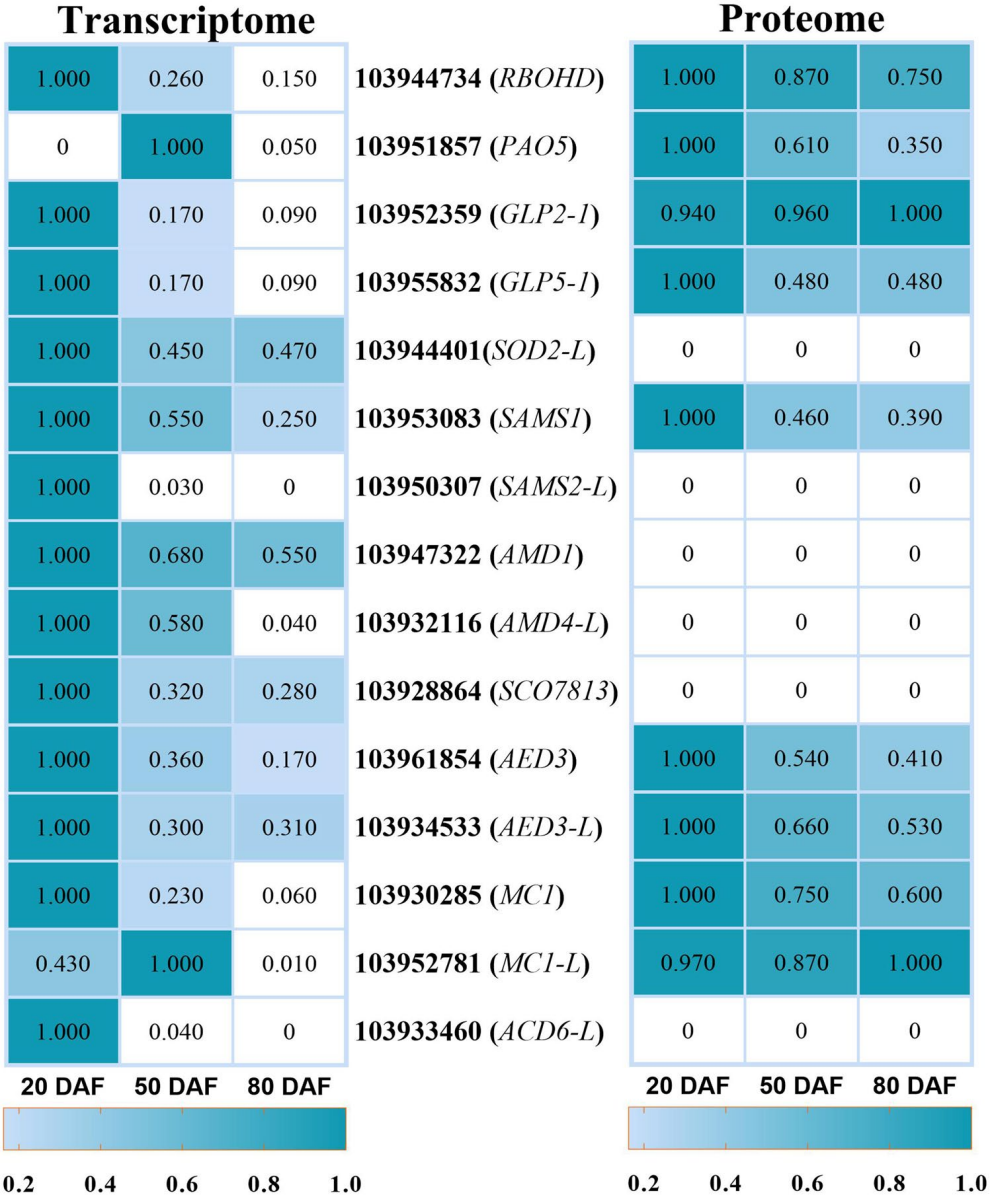
Other genes and proteins involved in apoplastic ROS production, SAM metabolic processes and PCD. The heat maps were produced using standardized figures that were transformed to a value between 0.0 and 1.0 by the min–max normalization method. In the proteomic data, the value 0 for all three periods indicates that no corresponding protein was detected.

To identify essential regulators and pathways involved in stone cell formation in Korla pear fruit, transcriptomic and proteomic analyses were performed via RNA sequencing (RNA-seq) and tandem mass tag (TMT) techniques. A total of 42,893 transcripts and 7904 proteins were identified, of which 24,268 genes and 1011 proteins showed differential expression during the development time course. Correlation analysis showed that approximately 13% (1010/7904) of the proteins correlated with DEGs, including 700 DAPs. Of the 1011 DAPs, 311 DAPs (30.8%) were not correlated with DEGs (Fig. 2A). Stone cells are formed by secondary thickening of parenchyma cell walls, and SCWs mainly consist of cellulose, hemicellulose, and lignin. Therefore, in this work, we closely focused on the DEGs and DAPs that were enriched in SCW-related pathways, including those associated with lignin biosynthesis (94 genes and 31 proteins), cellulose and xylan biosynthesis (46 genes and 18 proteins), S-adenosylmethionine (SAM) metabolic processes (10 genes and 3 proteins), apoplastic ROS production (16 genes and 2 proteins), and cell death (14 genes and 6 proteins) (Fig. 2B). Here, we list only the genes and proteins with greater differences in expression during the critical stage of stone cell differentiation (Figs. 3, 4, 5); information on all DEGs and DAPs in these pathways can be found in Supplementary Data S1-S3. Fifteen genes were randomly selected from among the DEGs for qPCR to validate the RNA-seq results. As shown in Fig. S1, the validity of the RNA-seq data was demonstrated by the high agreement between the RNA-seq data and qPCR data.

### DEGs and DAPs involved in lignin biosynthesis

The omics data revealed 94 DEGs and 31 correlated DAPs enriched in the lignin synthesis pathway in Korla pear pulp (Data S1). These genes and proteins encode upstream enzymes of the phenylpropanoid pathway, including phenylalanine ammonialyase (PAL), cinnamate-4-hydroxylase (C4H), *p*-coumarate 3-hydroxylase (C3′H), 4-coumarate: CoA ligase (4CL), *p*-hydroxycinnamoyl-CoA: quinate shikimate *p*-hydroxycinnamoyl transferase (HCT), caffeoyl shikimate esterase (CSE), and caffeoyl-CoA *O*-methyltransferase (CCoAOMT); enzymes in the monolignol-specific pathway, including cinnamoyl-CoA reductase (CCR), caffeic acid-5-*O*-methyltransferase (COMT), ferulate 5-hydroxylase (F5H), and cinnamoyl alcohol dehydrogenase (CAD); and enzymes involved in lignin polymerization (peroxidase (PRX) and laccase (LAC)). The majority of these genes showed peak expression at 20 DAF, after which the expression gradually decreased from 20 to 80 DAF, showing trends similar to the trend of lignin content during fruit development. Some transcripts, however, showed no differential expression from 50 to 80 DAF, indicating that lignin accumulation mainly occurred at the early stage of fruit development. Interestingly, the expression patterns of some transcripts encoding PAL (one transcript), C4H (two transcripts), 4CL (two transcripts), HCT (one transcript), CCR (five transcripts), CAD (two transcripts), CSE (two transcripts) and PRX (five transcripts) showed the opposite trend, indicating that they may be involved in the biosynthesis of other secondary metabolites involved in the phenylpropanoid pathway. However, the expression levels of these genes were almost negligible compared with those of the genes positively related to lignin content.

### DEGs and DAPs associated with cellulose biosynthesis

Cellulose is the most abundant component of SCWs. Twenty-nine DEGs in the transcriptome profile were assigned to cellulose biosynthesis, while 7 DAPs were annotated to the same process in the proteomic data (Fig. 4). UDP-glucose is the direct precursor of cellulose. Therefore, pathways that lead to UDP-glucose production can potentially have an impact on cellulose biosynthesis. UDP-glucose pyrophosphorylase (UGP) and sucrose synthase (SuSy) are the main enzymes that have been shown to produce UDP-glucose in plants^15^. Although we did not find any differentially expressed genes encoding UGPs, four DEGs and one DAP were found to encode sucrose synthase (SuSy), and their expression pattern was consistent with the trend of cellulose content, indicating that SuSy is tightly associated with cellulose biosynthesis. The hexose phosphate pool can also affect the UDP-glucose pool size. The biosynthesis of sucrose is inhibited by the feedback regulation of fructose, and fructokinase (FRK) phosphorylates fructose to produce fructose-6-phosphate. According to our omics data, 4 DEGs and 2 DAPs that encode FRK were selected. The expression of all of these genes and proteins was similar to that of *SuSy* during the experimental period, indicating that the synthesis of cellulose may be affected by FRK through reducing intracellular fructose pools, which can modulate SuSy activity and UDP-glucose production.

Cellulose synthase (CesA) uses UDP-glucose as a substrate for the biosynthesis of cellulose chains. In our study, we obtained a total of 21 functional genes and 4 proteins related to CesA enzymes, among which the expression patterns of the homologous genes of *Arabidopsis CesA4* (2 transcripts), *CesA7* (2 transcripts), and *CesA8* (2 transcripts) were the same as those of the lignin synthesis-related genes, following trends of lignin and stone cell contents during the experimental time course (Fig. 1). In addition, we screened 2 DEGs and 1 DAP annotated as COBRA-like 4 (*COBL4*) proteins. In *Arabidopsis*, *COBL4* is required for cellulose synthesis in secondary cell walls. The expression trends of these genes were similar to those of *CesA*s associated with secondary wall biosynthesis, implying that this protein is most likely involved in the assembly of cellulose synthase complexes. However, homologous genes of *Arabidopsis CesA1* (2 transcripts), *CesA2* (2 transcripts), *CesA3* (2 transcripts) and most cellulose synthase-like proteins (CSLs) (5 transcripts) showed the opposite expression trend.

### DEGs and DAPs associated with xylan biosynthesis

UDP-xylose is a nucleotide sugar required for xylan backbone elongation. According to our data, 25 DEGs and 13 DAPs were identified as xylan biosynthesis-related genes and proteins. All of these genes and proteins showed the same expression trends as the genes related to cellulose and lignin synthesis (Fig. 4). Among them, one gene with no associated protein identified encoded UDP-glucose dehydrogenase (UGD), which converts UDP-glucose to UDP-glucuronic acid; 6 genes and 5 associated proteins encoded UDP-xylose synthase (UXS), which is responsible for the conversion of UDP-glucuronic acid to UDP-xylose; 4 genes and 4 associated proteins encode xylosyltransferases (XylTs) needed for elongation of the xylan backbone; 4 genes with no associated proteins encoded glucuronosyltransferase (*GUX*), which is essential for the side chain addition; 6 genes and 3 associated proteins encoded glucuronoxylan 4-O-methyltransferase (GXMT) required for the glucuronic acid (GlcA) side chain methylation; and 3 genes (*IRX7*) with one associated protein required for the formation of the xylan reducing end sequence (XRES), which is speculated to be closely related to the initiation or termination of glucuronoxylan biosynthesis, were found^16^ (Fig. 4 and Data S2). As shown in Fig. 4, the expression levels of genes related to UDP-xylose formation (103,928,376, 103,928,424, 103,940,426, 103,948,402, 103,930,040 and 103,944,949)), elongation of the xylan backbone (103,947,711, 103,950,395, 103,942,677, etc.), and side chain methylation (*GXM*) were significantly higher than those of other xylan-related genes, implying their essential roles in xylan or glucuronoxylan synthesis. Most of the SCW- genes and xylan-related genes were preferentially expressed at 20 DAF, while primary wall-related genes were relatively abundant in the day-80 samples (Data S2). Taken together, these results suggested that the secondary thickening of parenchymal cell walls mainly occurred at the early stage of fruit development.

### Other secondary cell wall-related genes and proteins

Genes related to apoplastic ROS production (16 genes and 2 proteins), SAM metabolic processes (10 genes and 3 proteins), and cell death (12 genes and 6 proteins) were well represented among the candidate genes (Fig. 5 and Data S3). Apoplastic ROS can be used as oxidants in cell wall cross-linking and are required for lignin polymerization. ROS, which constitute a major stress factor, also play an essential role in apoptosis induction. Genes and proteins related to apoplastic ROS preferentially accumulated at 20 DAF, with markedly lower expression at later stages. For example, 3 genes and 1 associated protein annotated as respiratory burst oxidase homolog (RBOH) showed the highest expression at the early stage, after which the expression rapidly decreased during fruit development. Increased accumulation of transcripts encoding superoxide dismutase (SOD), polyamine oxidase (PAO), PRXs and germin-like proteins (GLPs) was also observed in early fruits.

The expression patterns of genes encoding enzymes related to the SAM metabolic process were similar to those of ROS-related genes (Fig. 5 and Data S3). Polyamines are important substrates for apoplastic H_2_O_2_ production, and polyamine generation also requires SAM to provide carboxyl propyl groups. Moreover, methyl groups required for the synthesis of cell wall polymers are also provided by SAM. Numerous genes related to programmed cell death (PCD) were also expressed in pear pulp and showed their highest expression at 20 DAF, which is consistent with the trends of genes that lead to ROS generation during fruit development (Fig. 5 and Data S3).

## Discussion

In pear fruits, some parenchyma cells undergo secondary thickening during fruit development, and lignin is deposited in the thickened cell walls to form stone cells^17–19^. Our results showed that the stone cell formation primarily occurred in the early fruits. Similar results have been reported for other pear varieties^1,2,9^. Cellulose, hemicellulose and lignin are the main components of secondary walls, and their proportions can vary among different plant species. The secondary walls of some special tissues consist mainly of one or two polymers. For example, cotton fibers are abundant in cellulose (> 90%), and phloem fibers, on the other hand, contain no lignin^4^. We found that pear stone cells were mainly composed of cellulose (52%), hemicellulose (23%), lignin (20%) and a small amount of polysaccharides (3%). Our results are also supported by several previous reports ^3,20^, indicating that stone cell formation is more likely to result from the differentiation of distinct cell types. In support of this, we found that many secondary cell wall-related genes and proteins (53 genes and 20 proteins) showed peak expression in early fruits, which was consistent with the change trends of stone cells and lignin.

The lignification process varies among different cell types; for instance, in TEs, cell apoptosis is a prerequisite for cell wall lignification, in which enzymes and/or substrates needed for cell wall lignification are provided by neighboring cells^12,21,22^. In contrast to that of TEs, lignification of fiber cells is carried out by cell-autonomous processes, in which cell apoptosis and cell wall lignification occur simultaneously^21,22^. The lignin composition varies between these cell types. In TEs, lignin is primarily composed of G-units, while S-lignin is abundant in the secondary walls of fibers^23,24^. It is a part of the folklore of plant biology that, like fibers, stone cells comprise sclerenchyma cells. Supporting this theory, in our previous work, we found that sinapyl alcohol, which gives rise to S-units, is the dominant lignin monomer in Korla pear fruit pulp during the whole period of fruit development^17^. These observations suggest that the differentiation of stone cells, which are types of sclerenchyma cells, must begin with the formation of a framework of secondary walls that are primarily composed of cellulose and hemicellulose.

Cellulose is the most abundant component of secondary cell walls. We found that the cellulose content accounted for more than half of the stone cell biomass, indicating that cellulose composes the skeleton of secondary walls of stone cells. Cellulose synthase (CesA) uses UDP-glucose as a substrate for the biosynthesis of cellulose chains^25,26^. Therefore, pathways that lead to UDP-glucose production can potentially have an impact on cellulose biosynthesis. SuSy and UGPs are the main enzymes that produce UDP-glucose in plants^15^. In hybrid aspen, inhibition of SuSy was shown to reduce carbon allocation to all secondary cell wall polymers; however, its overexpression increased only cellulose deposition in hybrid poplar^26,27^. In accordance with these findings, in our work, several genes (103950289, 103940505, 103940043 and 103964096) encoding SuSy showed peak expression at 20 DAF, after which their expression decreased more than 20-fold at 80 DAF. The change trends were consistent with those of the cellulose content, indicating that SuSy is tightly associated with the processes o cellulose biosynthesis. In our previous research, we found that SuSy showed higher enzymatic activity in early fruits, and SuSy activity decreased first but then increased gradually 60 DAF. Accordingly, the sucrose abundance increased slowly at the later stage and began to increase at 60 DAF^28^, suggesting that, in early fruits, sucrose is mainly used for cellulose synthesis. The hexose-phosphate pool can also affect the UDP-glucose pool size^29^. The biosynthesis of sucrose is inhibited by feedback regulation of fructose, and FRK phosphorylates fructose to produce fructose-6-phosphate^29^. We identified 4 DEGs and 2 DAPs that encode FRK and whose expression was similar to that of the *SuSy* gene during the experimental period, indicating that the synthesis of cellulose may be affected by FRK by reducing intracellular fructose pools, which can modulate SuSy activity and UDP-glucose production^15,29,30^.

In our study, *CESA* genes showed differential expression throughout the experimental time course. As expected, homologs of *AtCESA*s that encode CESAs in *Arabidopsis* and that are essential for secondary cell wall synthesis (*AtCESA4*, *AtCESA7* and *AtCESA8*)^31,32^ showed peak expression in early fruits, and the expression levels gradually declined at the later stage. In contrast, homologous genes of *AtCESA*s required for primary wall cellulose synthesis (*AtCESA1*, *AtCESA2*, and *AtCESA3*) in *Arabidopsis*^31,32^ showed the opposite expression trends. In addition, associated proteins of *AtCESA7* and *AtCESA8* were also found to be differentially expressed during the experimental time course and showed the same expression patterns as their corresponding genes did. These genes were reported to constitute secondary wall CesA complexes^33–35^. In addition, studies have shown that cesa4, cesa7, and cesa8 mutants have severe cellulose defects and reduced secondary wall thickness^31,32^. The same results were reported in rice, *Brachypodium* and *Populus*^34–36^, implying that *CESA* genes are required for secondary cell wall cellulose synthesis during the stone cell differentiation process.

Hemicellulose is another main component of pear stone cells, accounting for 23% of stone cell biomass. Xylan, a major component of hemicellulose, is essential for secondary wall strength ^37^. UDP-xylose is a nucleotide sugar required for xylan backbone elongation. Serial actions of UGD and UXS form UDP-xylose. Genes encoding these two enzymes showed relatively high expression in early fruits. The expression levels of *UXS*s were more than 100-fold higher than those of *UGD*s, indicating that UXS is the primary enzyme responsible for UDP-xylose. This is supported by the evidence that antisense downregulation of *UXS* resulted in fewer xylose-containing polymers and showed altered organization and reduced xylan phenotypes^38,39^.

Several enzymes have been identified to be involved in the elongation of the xylan backbone and its side chain addition. Genes (*IRX10*, *IRX15*, *IRX15-L*) encoding these enzymes and their associated proteins responsible for the synthesis of secondary cell wall xylan^40,41^ showed peak expression in early fruits, after which their expression decreased at the later stage. In *Arabidopsis*, all *irx* mutants have severely decreased xylan contents, chain length, and secondary wall thickness^40–44^. Interestingly, there is no significant reduction in xylan: XylT activity in the *irx15* and *irx15-L* mutants, and the *irx15 irx15-L* double mutant replaced GlcA with the meGlcA xylan side chain. Therefore, *IRX15* and *IRX15-L* define a new class of genes involved in xylan biosynthesis, but their function remains unknown^45–47^.

Another group of genes involved in the addition of GlcA side chains and side chain methylation, such as *GUX*s and *GXMT*s, showed peak expression in early fruits but were expressed at low levels at the later stages. These results are supported by evidence that *GUX* and *GXMT* are preferentially expressed in secondary thickening cell types, and *gux* and *gxmt* mutants are also found with a lack of GlcA or methylated GlcA in xylan side chains and reduced secondary wall thickness^48–50^. *IRX7* is required for the biosynthesis of XRES^51–53^. In accordance with this, we screened three DEGs (and one DAP) that were annotated as probable *IRX7*s and whose expression was substantially upregulated at the early stage and downregulated at the later stage, indicating that these genes are essential for xylan synthesis. Notably, mutations in XRES-related genes does affect either XylT or GlcAT enzyme activity, despite the loss of XRES^45,46^.

Lignin composes approximately 20% of stone cell biomass (Fig. 1). In previous studies, lignin-related genes were associated with the development of xylem and fibers in stems^54,55^. Similarly, in the present study, more than 90 genes and 31 proteins related to lignin biosynthesis were differentially expressed during pear fruit development (Data S1). The majority of these genes showed peak expression in early fruits and were positively correlated with lignin content, but some transcripts showed the opposite expression trends. However, the expression levels of the genes that were positively regulated with lignin content were overall much higher than those of negatively regulated genes, suggesting that these genes are essential for lignin biosynthesis during stone cell differentiation in pear fruits. Lignin-related genes have been studied extensively in different pear varieties^1,2,9,10,17,56^. Therefore, the role of these genes during lignin accumulation in pear pulp is not discussed in detail here. Instead, we mainly focused on the genes involved in lignin polymerization. After their biosynthesis, lignin monomers must be transported to the cell walls, where they are oxidized for polymerization^57–60^. Laccase and peroxidase are responsible for the oxidation of monolignols. In this work, the expression profiles of all *LAC* genes and the majority of *PRX* genes were similar to those of monolignol biosynthesis-related genes. Although some *PRX* genes showed the opposite trend, their expression levels were nearly negligible during the entire period compared to those of upregulated *PRX*s at the early stage. *LAC*s are reported to be coregulated together with monolignol synthesis-related genes and secondary cell wall formation-related genes^46,61^. We found 6 of 17 *LAC* genes, three transcripts of *LAC4-L*, two transcripts of *LAC17*-*L* and one transcript of *LAC2-L* whose expression was 10- to 150-fold higher than that of the other transcripts, indicating that these are the core *LAC*s for the formation of monolignol radicals. Studies have shown that the *lac4 lac17* double mutant presents a phenotype involving reduced lignin content, and the *lac4 lac11 lac17* triple mutant presents a phenotype involving severe growth defects and a loss of lignification of root vessels^61,62^. In model plant species, downregulating or silencing *PRX*s (*PRX2*, *PRX3*, *PRX25*, *PRX60*, *PRX71*, *PRX72*, etc*.*) resulted in reduced lignin accumulation and altered lignin composition^60–63^. Consistent with these findings, the results of our study showed that six transcripts of *PRX*s (two *PRX12-L*, two *PRX42-L*, one *PRX25* and one *PRX72-L*) were expressed markedly higher (up to several hundred times) than other transcripts were throughout the experiment. The abundance of the associated proteins *PRX12-L* and *PRX72-L* was also 10- to 100-fold higher than that of other associated proteins, suggesting their importance for lignin polymerization in Korla pear.

In addition to lignin monomers, peroxidases and laccases require H_2_O_2_ and O_2_, respectively, to form monolignol radicals. Our study showed that ROS-related genes and their encoded proteins were more abundantly expressed at the early stage and downregulated at the later stage. Specific ROS, such as H_2_O_2_, can be used as oxidants in cell wall cross-linking or as signaling molecules for controlling various biological processes^64,65^. It has been reported that scavenging H_2_O_2_ results in a reduction in lignification in the Casparian strip and TEs^21,66,67^. Together, these findings indicate that apoplastic ROS play an important role in the lignification process. H_2_O_2_ is also a key modulator of PCD, which is essential for the differentiation of TEs, fibers, etc.^68^ The present study also showed several highly expressed cell death-related genes and proteins (*AED3-like*, *MC1-like*, *ACD11-like*), and their expression patterns were similar to those of ROS-associated genes.

In conclusion, the differentiation of stone cells primarily occurred during cell division period of pear fruits, and no longer differentiated in the later stage of fruit development under the normal growth condition. Our results indicate that cellulose, hemicellulose, and lignin were the main components of stone cells. In addition to lignin synthetic genes, many other genes related to secondary wall development, cell apoptosis and ROS production also highly expressed during the critical period of stone cell differentiation, suggesting that stone cell formation in pear fruits required synergistic regulation of lignin synthetic genes and other genes involved in other SCW-related pathways.

## Methods

### Plant materials and sampling period

Fruits of Korla pear were collected from 20-year-old pear trees grown in an orchard in Korla (Xinjiang, China). The fruits were sampled at 20, 50 and 80 days after flowering (DAF), respectively. Stone cells are commonly present in all pear varieties. The differentiation of pear stone cells primarily occurs during cell division period of pear fruit development which usually takes place within 50 DAF, and after then, the stone cells are no longer differentiated in the later stage of fruit development under the normal growth condition. Therefore, the selected experimental periods in this study can represent the prime, late and stationary stages of stone cell differentiation in Korla pear fruits, respectively. At the early stages (20 DAF and 50 DAF), each biological replicate consisted of fifteen individual fruit samples collected from five independent trees, and ten fruits were collected for each biological replicate at the later stage. The fruit skins were peeled with a peeler, and fruit pulps were sampled, frozen in liquid nitrogen, ground into a powder, and stored at -80 °C until further analysis. The same batch of samples was used for transcriptomic, proteomic, physiological and biological analyses.

### Stone cell separation and structural carbohydrate detection

The rough-skin phenotype is not apparent for early fruits and gradually appears at the later stage of fruit development (late July to early August). Thus, stone cells were separated from 5 kg of rough-skin fruits at 100 DAF (August 15) using the method described by Brahem et al. (2017)^3^. The stone cells were collected, oven dried, and ground into a uniform powder. The ground powder was used for analysis of secondary cell wall components. The lignin content was determined by the Klason method^69^, the hemicellulose content was determined via 2% hydrochloric acid hydrolysis with the DNS method^70^, and the cellulose content was determined by the anthrone-sulfuric acid colorimetry method^70^. The polysaccharide content was tested by the phenol–sulfuric acid method^71^.

### Stone cell staining and apoptosis detection

Fruit pulps were cut into appropriate sizes and fixed with FAA fixative solution (formalin:glacial acetic acid:ethanol (90%) = 5:5:90, v/v). The preparation and tissue staining of paraffin sections were performed using the phloroglucinol method described by Tao et al. (2009)^2^. The same paraffin sections were used for apoptosis detection. Apoptosis was detected by TUNEL (terminal deoxynucleotidyl transferase-mediated dUTP nick-end labeling) assay kits from Shanghai Beyotime (www.beyotime.com). Briefly, paraffin sections were heated for 2 h at 65 °C in an oven and then dewaxed in xylene for 10 min. The tissues were then hydrated sequentially in a 100%, 100%, 95%, and 80% ethanol series and purified water for 5 min. The slices were then transferred to a wet box, 50 µg/ml proteinase K working solution was added to each sample, and the reaction was carried out at 37 °C for 30 min. The splices were then washed thoroughly with phosphate buffer solution (PBS; pH 7.2–7.4) 3 times, each for 5 min. During this step, protease K was washed away; otherwise, it strongly interfered with the subsequent marking reaction. The PBS around the tissue was absorbed with blotting paper, and a sufficient amount of TUNEL test solution was added to each glass slide. The slides were then incubated at 45 °C in the dark for 2 h, after which they were washed thoroughly with PBS 3 times for 5 min each. The PBS around the tissue was absorbed with blotting paper. Apoptosis was observed under a fluorescence microscope after sealing the slices with antifluorescent quenching agent. The regulated dead cells showed green fluorescence.

### Transcriptomic sequencing

Transcriptomic sequencing was performed at Novogene Bioinformatics Technology Co., Ltd. (Beijing, China). In brief, total RNA was isolated by the use of TRIzol reagent, and the purity, concentration and integrity of the RNA were determined. Once the RNA samples were qualified, oligo-dT beads were used for the isolation of mRNA. The isolated mRNA was fragmented into short sections and used for the synthesis of first-strand cDNA. The synthesis of second-strand cDNA was performed by use of dNTPs, DNA polymerase I and RNase H. The purification of double-stranded cDNA was carried out by using AMPure XP beads, and then the purified double-stranded cDNA was used for end reparation, polyadenylation and ligation of adapters. After size selection with AMPure XP beads, PCR amplification was performed, and the product was purified using AMPure XP beads for library construction. Finally, the library consisting of 2 × 150 bp paired-end reads was sequenced on an Illumina HiSeq P150 sequencer (Illumina, San Diego, CA, USA). The raw data were cleaned by removing reads with adapters, reads of low quality (> 50%) or reads with a high proportion of unknown bases (> 10%) and then assembled with the Trinity software package. Annotation of transcriptome sequences was performed by using the following public databases: the Nr (NCBI nonredundant protein sequences), Nt (NCBI nucleotide sequences), Pfam (Protein family), KOG/COG (eukaryotic Ortholog Groups and Clusters of Orthologous Groups of proteins), Swiss-Prot (a manually annotated and reviewed protein sequence database), KEGG (Kyoto Encyclopedia of Genes and Genomes)^72^ and GO (Gene Ontology) databases. The mapped read numbers were transformed into FPKM (fragments per kilobase of transcript sequence per million base pairs sequenced) values for gene expression quantification and differential expression analysis. KEGG pathway analysis was conducted, and the corrected *P*-value cutoff was set at 0.05. The sequencing data have been deposited in the NCBI Sequence Read Archive (SRA) database under the accession number PRJNA588520.

### Proteomic sequencing

Proteomic sequencing was performed at Novogene Bioinformatics Technology Co., Ltd. (Beijing, China). In brief, the concentrations of proteins were determined using the Bradford assay, and 100 µg of protein from each replicate was digested with Trypsin Gold (Promega, Madison, WI) at 37 ℃ for 16 h. The peptides were then dried by vacuum centrifugation, and the desalted peptides were labeled with TMT6/10-plex reagents (TMT6/10plex Isobaric Label Reagent Set, Thermo Fisher) according to the manufacturer’s instructions. After labeling, the samples were mixed and lyophilized. The TMT-labeled peptide mix was fractionated using a C18 column (Waters BEH C18 4.6 × 250 mm, 5 µm) on a Rigol L3000 HPLC instrument. Eluent was collected every minute and then combined to generate 15 fractions. Shotgun proteomic analyses were performed using an EASY-nLC 1200 UHPLC system (Thermo Fisher) coupled to an Orbitrap Q Exactive HF-X mass spectrometer (Thermo Fisher) operating in the data-dependent acquisition (DDA) mode. Peptides were separated on a Reprosil-Pur 120 C18-AQ analytical column (15 cm × 150 μm, 1.9 μm). Full MS scans from 350 to 1500 m/z were obtained at a resolution of 60,000 (at 200 m/z), with an AGC target value of 3 × 10^6^ and a maximum ion injection time of 20 ms. From the full MS scan, a maximum number of 40 of the most abundant precursor ions were selected for higher energy collisional dissociation (HCD) fragment analysis at a resolution of 15,000 for TMT6-plex (at 200 m/z) or 45,000 for TMT10-plex (at 200 m/z), with an automatic gain control (AGC) target value of 1 × 10^5^, a maximum ion injection time of 45 ms, a normalized collision energy of 32%, an intensity threshold of 8.3 × 10^3^, and a dynamic exclusion parameter of 60 s. Differentially accumulated proteins (DAPs) were selected based on p < 0.05 and |log2FC|> 0.585. To identify the most important biochemical metabolic pathways and signal transduction pathways involved in the DAPs, KEGG analysis was performed. GO enrichment analysis of DAPs was also performed to identify the major biological functions.

### Quantitative real-time PCR validation

Fifteen genes that were mapped to the lignin, cellulose and xylan biosynthesis pathways were randomly selected for quantification via real-time PCR. The sequences of the primers used were designed using the Oligo 7.0 software package. The specificity of the primer sets was examined by running the PCR products on agarose gels to ensure single-band amplification. Approximately 1 μg of total RNA was used for the synthesis of cDNA with RR037A reverse transcriptase (Takara, USA). qRT-PCR was performed in a 20 μl system consisting of 1 μl each of both forward and reverse primers, SYBR Green Real-Time PCR Master Mix (Invitrogen, USA) and 1 μl of cDNA template on a Step-One Plus Real-Time PCR System (Applied Biosystems, USA). The cycling conditions were as follows: 95 °C for 15 min followed by 40 cycles of 95 °C for 10 s, 58 °C for 20 s, and 72 °C for 20 s. Each sample comprised three individuals as replicates. The expression level of each gene was normalized to that of tubulin, and the relative gene expression level was calculated with the 2^-ΔΔCt^ method^73^.

### Statistical analysis

The statistical significance of differences in this study was analyzed using one-way ANOVA followed by Tukey’s post hoc test with a significance level of 0.05 (p < 0.05) (GraphPad Prism 7.0 software for Windows).

### Ethics approval and consent to participate

The fruit samples were collected in an orchard at Korla (Xingjiang, China) with the permission of the local grower. The experimental research on plants, including collection of plant material, was complied with institutional, national, or international guidelines. And field studies were conducted in accordance with local legislation. And also comply with the Convention on the Trade in Endangered Species of Wild Fauna and Flora.

## Supplementary Information

Supplementary Information 1.

Supplementary Information 2.

Supplementary Information 3.

Supplementary Information 4.

Supplementary Figure

## Data Availability

① The raw data of transcription sequencing generated during the current study are available in the NCBI Bioproject repository (https://www.ncbi.nlm.nih.gov/bioproject/PRJNA588520). ② The raw data of proteome sequencing generated during the current study are available in the iProX repository (https://www.iprox.org/page/project.html?id=IPX0002167000). It also can be found in the repository of ProteomeXchange (http://proteomecentral.proteomexchange.org/cgi/GetDataset?ID=PXD018829). ③ All data analyzed during this study are included in this published article (and its supplementary data files).
