# Supplementary figures and images for: Integrated transcriptomic and proteomic analysis reveals the complex molecular mechanisms underlying stone cell formation in Korla pear
Source: Sci Rep. 2021 Apr 8;11:7688. doi: 10.1038/s41598-021-87262-3 (PMC8032765; doi:10.1038/s41598-021-87262-3)

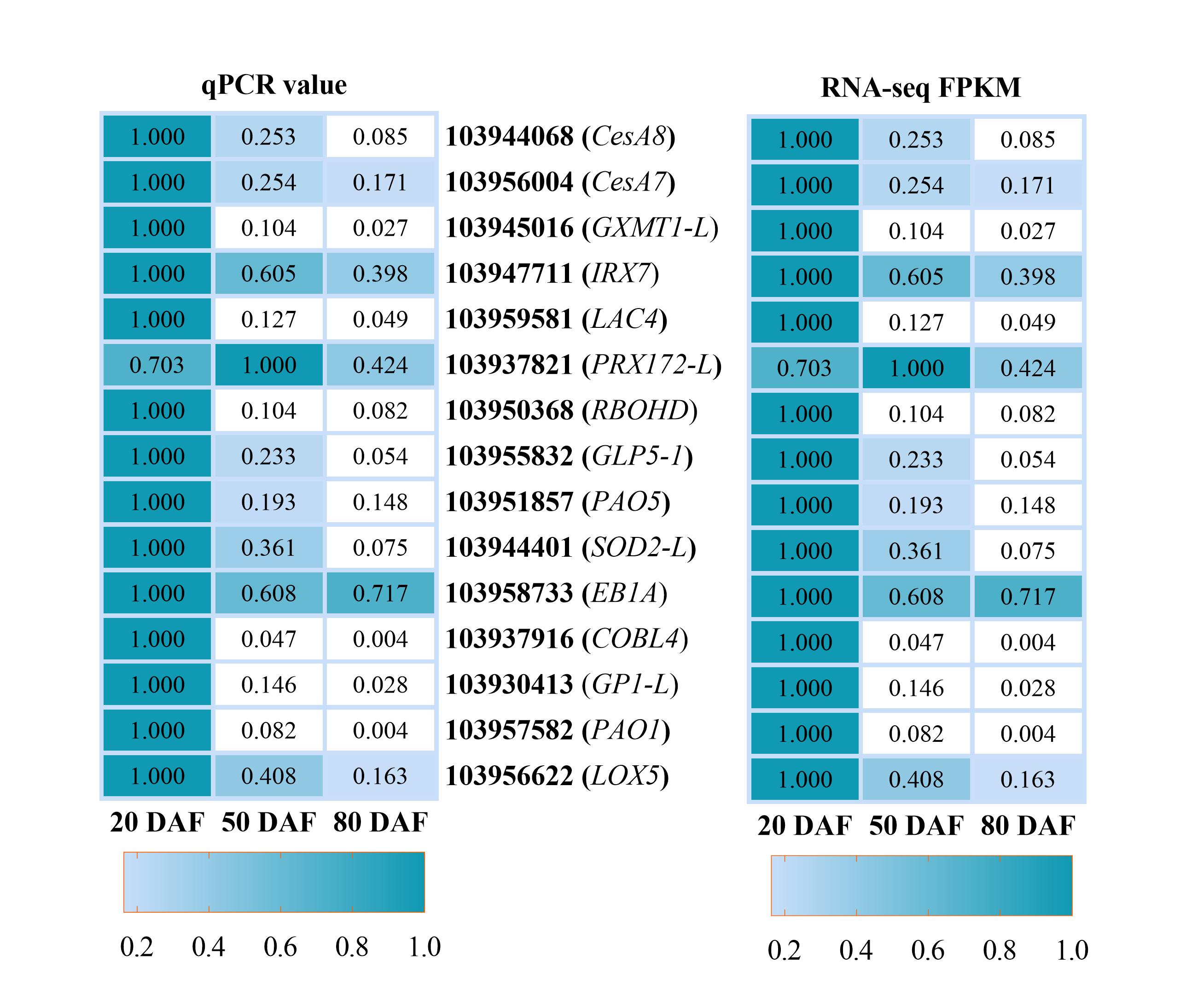

Supplement: Supplementary file 5 — Supplementary Figure [file 41598_2021_87262_MOESM5_ESM.jpg]
